# Living with sibling’ drug use. Bereaved siblings’ family stories

**DOI:** 10.1080/17482631.2023.2240576

**Published:** 2023-07-28

**Authors:** Sari Kaarina Lindeman, Lillian Bruland Selseng, Lennart Lorås, Aina Helen Løberg

**Affiliations:** Faculty of Health and Social Sciences, Western Norway University of Applied Sciences, Bergen, Norway

**Keywords:** Siblings, family relations, problematic drug use, drug-related death, narrative research

## Abstract

Family members’ problematic drug use is challenging for siblings affecting their well-being and their relationships within the family. Research about siblings living with brothers or sisters’ problematic drug use and research on bereaved siblings’ experiences indicates that life situations and support needs for both minor siblings and adult siblings can easily be overlooked, both in practice and in research. This article contributes to this knowledge gap by examining how siblings provide meaning to their sibling’s drug use problem and how they position themselves and other family members accordingly. Qualitative semi-structured interviews were used for data collection, and fourteen bereaved siblings were interviewed. A narrative thematic analysis was chosen, and four themes were generated. These four themes, (1) Surviving difficult family life, (2) The relationships in continuous change, (3) It’s worse for the parents, and (4) “We”, as a synonym for the family, are presented in this article. Our findings demonstrated how complex and multifaceted siblings’ stories about living with their brothers or sisters’ ongoing drug use are. This study calls for more attention to siblings’ situations. Siblings’ lives are affected by their brothers or sisters’ problems, and siblings should also be involved in routine support and treatment practices.

## Introduction

This study explores bereaved siblings’ stories about how siblings’ drug use problems affected their family relations. The literature has viewed sibling relationships as distinctive in many ways (Dunn, 1983; Rocca et al., 2010; Spitze & Trent, 2018). Sibling relationships can have multiple elements, such as friendship, biological attachment, shared family stories, and equal status (Connidis, 2010; Spitze & Trent, 2018; L. White, 2001). Sibling relations can also be the longest-lasting relationships in an individual’s life, starting from birth and continuing until death (Seltzer et al., 1991). That means that siblings may “bear common witness to more of the changes made across the life course than most other family ties” (Connidis, 1992, p. 972). These changes can be joyful events such as a sibling falling in love, giving birth or succeeding in a career, but also seeing a sibling going through demanding life changes like divorce, bereavement, or illness. A growing body of research has documented sibling relationships’ developmental and supportive significance across the lifespan (Conger & Kramer, 2010; Dunn, 2018; Feinberg et al., 2012; Lamb, 2014). However, little is known about how individual sibling relationships change in response to life events. The burden on an adult sibling who experiences their siblings’ serious mental illness or substance use problems doesn’t receive a great deal of attention, both in research and social and healthcare practices (Schmid et al., 2009; Smith-Genthôs et al., 2017).

An article from Orford et al. (2010), which summarize decades of research on the consequences of problematic substance use for family members, and two systematic literature reviews (Lindeman et al., 2021, 2023), shows how family members’ problematic substance use overwhelmingly impacts family life. Families often experience a lack of support because their complex landscape of needs has not been understood (Lindeman et al., 2021, 2023; Orford et al., 2010). The core experience of being a family member of a substance-using person is influenced by the characteristics of the relationship and the ages of each family member (Orford et al., 2010). Research about the experiences of next of kin in the psychiatric and substance use field has mainly focused on parents or spouses (McAlpine, 2013; Schmid et al., 2009). A sibling relationship differs from a relationship between a parent and child or partners. Dunn (1983) points out that sibling relationships include the complementary interactions typical of adult-child relationships and the equal and mutually influential interactions often typical for peers.

Due to the sibling relationship’s unique character, there is reason to believe that siblings may have different struggles and needs for help than parents and partners. When a brother or sister has substance use problems, a sibling’s story is often influenced by the whole family (Howard et al., 2010). The strain of problematic substance use impacts the family’s entire life, from everyday activities to future dreams (Lindeman et al., 2021), and therefore also affects siblings’ relationships within the family. Several qualitative studies have described that parental attention and energy is focused more on the sibling with substance use problems rather than the sibling without (Barnard, 2005; Gabriel, 2017; Incerti et al., 2015; Schultz & Alpaslan, 2016). Schultz and Alpaslans’s (2016) study with 28 adult siblings as participants described how functioning in families rotated around the child with problematic substance use, and parents made continuing attempts to get the child into treatment and to prevent new episodes of substance use. In Gabriel’s (2017) doctorate thesis, participants, six adult siblings, described how their sibling’s problematic substance use made them feel forgotten or overlooked within the family. Also, IIncerti et al. (2015), in a qualitative study with 13 adult siblings, concluded that siblings’ problematic substance use negatively affected not only the participants’ sibling relationship but also their relationship with their parents. Divided parental attention was also described both by parents (20 parents) and siblings (20 siblings under age 25) in Barnard’s (2005) study.

In substance use literature, sibling stories describe a loss of important relations and normal childhood (Gabriel, 2017; Howard et al., 2010; Tsamparli & Frrokaj, 2016). Howard and co-authors, a self-biographic article written by seven sisters of current or former brother or sister with problematic substance use, described how it was much about what does not happen (e.g., meaningful relating) as what does happen (e.g., chaos) (Howard et al., 2010, p. 465). Tsamparli and Frrokaj (2016) aimed with a mix-method study to examine the quality of sibling relationships in families with a sibling with problematic substance use and compare the relationship to families with a sibling with no use. Thirty-six families participated in the study, and 20 adult siblings with a brother or sister were interviewed. The authors described how siblings experienced a loss and mourning while their sibling was alive. Siblings can also experience anger, as J. M. Ólafsdóttir (2020) described in her mixed methods doctorate work concerning the impact of substance use disorder on the family system. Siblings participating in that study tended to express more hostile feelings than, for example, parents and adult children. Feelings siblings described could also be apathy, fading hope that their sibling could be able to make changes and also rage over the damage their sibling had perpetrated on the family (J. M. Ólafsdóttir, 2020; J. Ólafsdóttir et al., 2021). Siblings witnessed the negative impacts on other family members from one member’s drug use, as a qualitative doctorate thesis with 25 adult siblings (McAlpine, 2013) and a qualitative study with 14 siblings (Løberg et al., 2022) explained. Worries about how brothers and sisters substance use was impacting parents’ or caregivers’ well-being, mental health and physical health were part of siblings’ life, as Swinton (2020) described in her qualitative doctoral thesis, which involved interviews with 24 siblings aged between 17 and 30 years. Siblings were often protective and defensive of their parents (Barnard, 2005; Løberg et al., 2022), feeling responsible for their happiness (J. Ólafsdóttir et al., 2020).

Orford (2017, p. 14) pointed out that the greater the accumulated burden of hardships a family member is experiencing, the more challenging it is to cope with a relative’s problematic substance use. For some siblings, their family’s story may also be a history of difficult childhood or childhood maltreatment. The Adverse Childhood Experience Questionnaire (ACE-Q) has shown the link between adverse childhood experiences and adult mental and physical illnesses (Felitti et al., 1998; Zarse et al., 2019). The possibility of problematic substance use increases with childhood abuse and other adverse childhood experiences (ACEs) (Dube et al., 2003; Zarse et al., 2019). Higher ACE predicts the earlier start of illicit drug use and the adverse psychiatric consequences associated with drug use (Zarse et al., 2019). Some siblings grow up in the same families, with the same childhood experiences as their substance-using siblings. As a result, they may experience the accumulated burden of their siblings’ drug use and the consequences of a difficult childhood.

We argue that understanding the substance use problem, the family situation, and family members’ role in it is central to understanding siblings’ experiences. Earlier research has concluded that family members’ problematic substance use is challenging for siblings. However, we know little about how siblings give meaning to their siblings’ problems and the family’s role. This article contributes to this knowledge gap by examining how siblings provide meaning to their sibling’s drug use problem and how they position themselves and other family members accordingly. Research about siblings living with brothers or sisters’ problematic substance use (Smith-Genthôs et al., 2017) and research on bereaved siblings’ experiences (Gilmer et al., 2012) often refer to them as a forgotten group and indicates that both minor siblings and adult siblings can easily be overlooked. Research on the experiences of bereaved siblings after drug-related deaths has also shown that life before death strongly influences the time after death (Titlestad & Dyregrov, 2022). Knowledge about siblings’ stories and experiences is important to understand better what help and support needs siblings have, both while living with brothers and sisters ongoing substance use and as bereaved after drug-related deaths.

Keeping in mind the complexities and diversities in siblings’ stories, we wish to create more knowledge about how siblings tell their stories of the impact on their family relationships when problematic drug use is present. We are interested in how the siblings make sense of their experiences through their stories and in studying how their stories affect their family relationships. Therefore, we have chosen a narrative research focus for this article. The central idea in narrative research is that people create meaning in life by organizing their experiences as stories (M. White et al., 1990). The purpose of narrative research is not to generalize but to embrace the contextual, diverse, and nuanced (Blix et al., 2013). These stories impact how people experience themselves, their relations, and their possibilities of action. As Frank (2020) wrote, stories are crucial mediators of what constitutes people’s reality. Analysis of siblings’ stories is an entree to gaining insight into how they assemble meaning for their experiences through stories. From a narrative perspective, every personal story will always be linked to local and cultural resources that culture and social relationships make available for people and that people use to help construct their personal stories (Gubrium & Holstein, 1998; Smith, 2016).

This study is part of a large Norwegian study called “The Drug Death Related Bereavement and Recovery Study” (The END-project’). The project’s main purpose was to contribute to a greater understanding of the consequences of drug-related death (DRD) for bereaved family members and friends and about what help from professionals and support from the social network they need. The current article focuses on the siblings’ stories about the time before death. The study aims to describe and explore how siblings assembled meaning about their family relationships when a brother or a sister had problems with illicit drugs. The study has the following research question: *How do bereaved siblings talk about the impact siblings’ drug use problems had on their family relationships*?

## Methods

This study has a qualitative, descriptive, and exploratory design. Qualitative semi-structured interviews were used for data collection, and fourteen bereaved siblings were interviewed. Narrative analysis was chosen to generate an understanding of how siblings talk about the impact of living with siblings’ problematic drug use on family relations.

### Ethical considerations and methodological issues

All procedures were conducted following the Declaration of Helsinki (The World Medical Association, 9. July 2018). This study was approved in February 2018 by the Norwegian Regional Committees for Medical and Health Research Ethics (reference number 2017/2486/REK vest).

Participation was voluntary, and the participants were informed that they could withdraw their consent at any time without suffering any consequences. All the participants were given the opportunity to choose the location for the interviews. Eleven of the interviews were done at the participants’ workplace or home, while three interviews were done in the interviewer’s office or hotel room. According to Dyregrov’s (2004) suggestions regarding research on vulnerable populations, care was provided to the participants. The main project leader was available to organize support for participants who wanted to speak to somebody after participating in the interviews. The interview data were treated confidentially. All identifying information was anonymized and stored on the research server at the university.

We have followed The CASP Checklists for qualitative research (CASP, 2019) to improve the transparency and wholeness of the research process. We have given a clear statement of the aim of this study and data collection and described distinctive characteristics of participants, such as the siblings and the deceased’s age and the time since death. As we consider it, the study design and analysis are appropriate to address the research aims.

Both interviews and the analysis will always be subjective to a certain extent. The authors’ backgrounds influence the analysis. All authors have broad clinical and research experience in health and social sciences. All authors are clinical social workers; the first, second, and third authors are also family therapists. All authors have experience as social workers in substance use and the mental health field. Although the presented stories are co-constructed by the interviewer and the siblings, it is the relationship between the bereaved siblings’ assembled meanings and their family relations that are prioritized in the analysis. We have tried to make our choices in this research transparent for the reader.

### Participants and recruitment

In 2018, drug-death bereaved family members and friends were recruited for the main project from all parts of Norway. Recruitment was facilitated through flyers sent to municipalities, media, and services. Participants were also recruited by snowball recruitment. It means that participants and collaborators recruited possible participants from their network based on this study’s inclusion criteria.

All informants in the current study were recruited after participating in the END study’s questionnaire survey. In questionnaires, information was provided about the participants relevant to obtaining a strategic and heterogeneous variety. In addition, participants had to meet the following inclusion criteria: experiences of having lost a sibling to a drug-related death. The time that had passed since they had lost their sibling ranged from 3–360 months.

Ten women and four men agreed to participate, ranging in age from 23 to 61 years. The participants came from all parts of Norway. The gender distribution among the deceased siblings was one woman and thirteen men. All participants were asked in the questionnaire how close they felt to their siblings at the time of death. Thirteen participants described their relationship with substance-using siblings as very close or close, while one of the informants marked it as not so close. To ensure their confidentiality, we have used pseudonyms in the presentation of the findings. More information about the participants can be found in Table I.

**Table I.** Participant information.

Gender of study participant’	women	10
	men	4
Age of study participant’	30–39	7
	40–49	4
	50–61	3
Highest education of study participant’	Upper secondary school	3
	University college/university	11
Gender of the deceased	women	1
	men	13
Age of the deceased	17–20	1
	21–30	4
	31–40	7
	41–50	2
Time interview conducted after the death of a sibling	19–27 months	3
	44–89 months	3
	116–155 months	5
	211–360 months	3
Closeness to the deceased at the time of death	Very close or close	13
	Less close	1
Was aware of siblings’ substance use before death	All participants	14
Siblings’ drug use before death	7–10 years	5
	11–20 years	6
	21–30 years	3

### Interviews

Qualitative semi-structured interviews were used for data collection. Participants talked about their experiences in interviews conducted by three of the authors (SKL, LL AL) between 24. June and 04. December 2019. The interviews lasted between 45 and 159 minutes (the average length was 125 minutes) and were transcribed verbatim. The interviews had a retrospective focus in which the participants were asked to look back on the time before their siblings died. The interview followed topics in the interview script. While answering, the participants moved thematically back and forth between stories about the past, thoughts of the present, and views of the future. Some of the participants wanted to talk a lot about the death of their siblings, but for others, bereavement was a minor part of the story.

### Narrative analysis

Narrative research involves many different methods of analysis (Riessman, 2008). Riessman suggests the typology of narrative analysis based on the distinction between four ways of handling and analysing narratives: thematic, structural, dialogic/performative, and visual. The analysis model implemented in this study was inspired by Riessman’s (2005, 2008) narrative thematic analysis. Narrative thematic analysis is a creatively adapted version of thematic analysis developed by Braun and Clarke (2014). In addition, Riessman (2008) points out that narrative thematic analysis is case-centred and that stories are the unit of analysis.

People’s experiences offer an endless supply of storable items, but integrating these items into a coherent account gives them meaning (Gubrium & Holstein, 1998). Gubrium and Holstein (1998) describe how local and cultural resources provide guidelines for how stories unfold, but they do not determine individual storylines. Instead, individual stories uniquely emerge as they are told, “just as available storylines are put to use, and biographical particulars are linked together in locally accountable ways” (Gubrium & Holstein, 1998, p. 166). Gubrium and Holstein (1998) present narrative linkage as a concept to understand how people make meaning of their life experiences. The narrative linkage is about how the storyteller gathers his or her interpretation by linking together different spheres of meaning in the context of lifelong experience (Gubrium, 2001). The meaning and coherence of a story are thus drawn as much from such “narrative linkages” as from the disparate items and available plots from which a story is composed. We have used narrative linkage as an analytic concept in our analysis.

The analysis process was completed in six phases:

1. To allow the individual units of meaning to emerge from the transcribed text of the interviews, the first author worked with a single interview at a time, reading and taking notes of possible narrative themes of siblings’ family relations. Then, in dialogue with the second author, these individual units of meaning started to emerge in the form of themes in each interview. Due to the study’s relational focus, we were particularly concerned about asking questions like “What is the story of the family about, how does this story link experiences about relations in the family” and “how does the participant create meaning about his/her relations to other family members?”
2. The first author summarized our interpretation of the essence of each participant’s story in short summaries, trying to keep in mind the family context for the stories. These narrative extracts are in this study called focused summary stories.
3. Reflexive thematic analysis, as presented by Braun and Clarke (2019), was conducted, and four themes were generated across the interviews. All authors participated in defining and naming themes. Four main themes generated from the analyses were: (1) Surviving difficult family life, (2) The relationships in continuous change, (3) It’s worse for the parents, and (4) “We”, as a synonym for the family. The occurrence of the themes in interviews is presented in Appendix I.
4. The focused summary stories of the “essence” of the stories were compared, inspired by narrative researchers such as Blix and co-researchers (2013). The first and second authors critically discussed the focused summary stories. Then, four sibling stories were chosen to present the themes because they most clearly illustrate the themes from the interviews and represent diversity and contextual knowledge about siblings’ experiences of family relationships. The four stories do not give an exhaustive presentation of the stories told.
5. The four selected sibling stories were presented and discussed with the other authors. Stories were labelled, illustrating the theme that emerged in each story: Marthe’s story: It’s about the whole thing. (Theme 1: Surviving difficult family life), Gretha’s story: a long history as a relative (Theme 2: The relationships in continuous change), Tom’s story: The history of mother’s burden (Theme 3: It’s worse for the parents), and William’s story: The history of we (Theme 4: “We”, a synonym for the family). These four stories presented in this article are constructed from the researchers’ retellings and interpretations of the participants’ stories to illustrate themes.
6. The last phase concerns the writing of the article. The first author took primary responsibility for the first draft of the various parts and sent it to the other three researchers for commentary and input. We have chosen to illustrate themes with focused summary stories close to the participants’ stories. The use of focused summary stories can increase and deepen our understanding of how bereaved siblings experience the impact of living with a brother or a sister with drug use problems on their family relations. It also follows the intention of the narrative analysis of considering each story as a whole and not as story fragments (Frank, 2012). However, this is our interpretation of the participants’ stories, and there were always other stories to tell. As Frank (2012, p. 16) wrote: “the narrative analysis gives increased audibility to some stories, recasts how different stories are understood, and neglects many stories. But one analyst’s neglect is another’s possibility, less cause for criticism than for appreciation. The dialogue always continues”.

## Findings

We present four themes, illustrated by focused summary stories identified from interviews illuminating various aspects of how siblings’ experiences are narrated. The themes are in sibling interviews intertwined but are analytically separated. We have labelled the four themes (1) Surviving difficult family life, (2) The relationships in continuous change, (3) It’s worse for the parents, and (4) “We”, as a synonym for the family. The quoted excerpts are slightly edited and translated into English, and speakers have been given pseudonyms.

### Theme 1: surviving difficult family life

Theme 1 constructs how siblings’ ongoing drug use is just one of the challenging conditions. The participants presenting this story described conditions of upbringing with multiple problems. In this story, the parents’ socioeconomic and psychosocial difficulties are made relevant to the life situation in the family. It also impacted how the story of life with a sibling was linked and told. Through retelling some of Marthe’s story, we illustrate some of the elements of this theme.

#### Marthe’s story: It’s about the whole thing

Marthe told the story of how she and her siblings grew up with their mother, who had alcohol use challenges. Her story is about the mother who had said several times that she wished that the sisters had never been born. The sisters’ relationship with their father is portrayed as difficult because being with their father resulted in sexual abuse. Marthe recounted that all three sisters had difficulties due to their upbringing and that both her sisters developed drug use challenges. The family lived in a transparent small town, and she related that everyone knew that her mother was drinking and both siblings had problems with drugs. Marthe creates a story about herself as a girl who fought not to be labelled as part of her family’s struggles. Marthe defined a clear difference between herself and the rest of the family:

I’ve always felt like the black sheep in the family. I was an angry child who was given liquid sedatives because I would scream like crazy. I would hit my head against the wall, but that’s how children express emotional pain.

In her story of surviving difficult family life, separating one problem from another is challenging. Marthe linked the story of her siblings’ developing drug use problems to their childhood experiences. She had a statement that if anyone should have helped them, they should have started when they were little. She narrated how overwhelming and complicated experiences can be difficult for outsiders to understand. In Marthe’s own words: “I don’t think I can clearly explain how much this affects your entire being.”

Marthe used the word “we” when talking about her siblings: “Considering our upbringing, we are used to fending for ourselves, comforting ourselves and looking after ourselves.” Linked to upbringing, she explained that all three siblings had a hard time, but they solved it in different ways:

It’s all about the big picture. There is a reason that my sisters started taking drugs, which I understand completely. I think a lot about whether things could’ve been different. I survived just fine and have become who I am today because of or in spite of it, and I’m quite happy with that. But I think a lot about my sisters, especially the oldest one who died. I can’t visit her grave without crying – not so much because she died, but more because of the life she led. The worst part is that my mum is buried in the same grave. Her being dead isn’t the sad part, but rather that she was never really a mother to us.

Marthe pointed out the difference between life situations in well-functioning families, where one family member dies of drug-related death, compared with the life situation where several family members live in drug use and then lose one member of the family member to a drug death.

Marthe’s story as a sibling is linked to surviving difficult and destructive conditions when she was growing up. She linked her sisters’ substance use to their complicated family history and held her parents responsible. She described how she was in continuous movement between distancing herself from her parents, protecting herself, and entering back into the family to solve problems for other family members. Marthe used formulations like: “choose to withdraw,” “choose to distance themselves,” and “choose to help.” Some of the choices seem to be more challenging than others. Marthe also used the narrative linkage about childhood hardship when discussing her future. She talked about the consequences of the care situation, which continued for generations. She explained that when the young children in the family are in demanding care situations, Marthe felt that she had to help “then I have to step in again.”

Marthe ended her story by emphasizing the perspective of the family member who has managed to live a life without drug use despite a demanding upbringing. She stated how she often felt that the focus and compassion, both privately and in the media, has been directed towards family members who have developed drug challenges. In her story, she has not been recognized but is needed when siblings and their children struggle. The story about strength and surviving culminated in Marthe saying that if she gets help from others someday, “it will probably make her feel weak because it will be such a defeat”.

### Theme 2: the relationships in continuous change

The theme “The relationships in continuous change” illustrates how the participants’ story of family life with a sibling with ongoing drug use problems is constructed as a long-term rollercoaster. Participants changing understanding of the sibling’s drug use problem is made relevant in these stories. They tell how the change process involved re-evaluating their resources for helping, resigning, and seeking new possibilities. The re-evaluation of the sibling relationship is linked to their relationships with other family members, their own lives, and other close relations. Their experiences are portrayed in Gretha’s story as a far-reaching and comprehensive process of change.

#### Gretha’s story: a long history as a relative

Gretha’s story extended over almost 30 years. Her story was linked to the development that occurs over time as a relative to a family member with ongoing drug use problems. As Gretha explained:

I had a little brother with a big drug problem for almost as long as I can remember. He was 41 years old when he died, and we had a long history together – with me as a relative, a sister, and him with his problems. I look back on it as a rollercoaster, from when he started using until my parents found out about substance use.

Gretha linked her story to different phases in her life where she positions herself differently from her brother’s life situation and, at the same time to other family members. The first part of Gretha’s story was about unsuccessful attempts to help. She explained how she tried to meet the expectations that she experienced other family members had for her to manage to help her brother, but she couldn’t do it. She creates the story about a daughter who wants to save her parents and brother but cannot manage it. Gretha related how she tried to be a perfect daughter and sister, resulting in a demanding double play. Gretha explained how helping her family members became a struggle:

I chose a helping profession, and looking back; I don’t think that was a coincidence. I couldn’t help my brother, but I wanted to be able to help others. I used quite a bit of energy trying to figure out how to help him. My mother suffered a great deal emotionally, and not only because of my brother. She often used me as her advisor. This put some of the blame for the problem(s) on me because I didn’t have the right advice to give.

Here Gretha linked her story and position in the family to understanding that she could not help her brother and parents. She created a picture of the deadlocked situation. On the one hand, her advice is expected because her family is presented as not dealing with her brother’s problems themselves, and when her advice does not help, she becomes an accomplice.

The middle part of the story is about difficult years. Gretha explained that all her unsuccessful attempts to help had consequences such as low self-esteem, guilt, and broken relationships with partners. Gretha stated that she felt that she was being suffocated. Finally, in what is presented as a turning point in her story, Gretha increasingly understood that she could not help her brother or mother and chose to move far from the family and her childhood town. She told this story as follows:

I wasn’t able to live my own life because there was always someone I felt guilty about or needed to help or offer support to. In the end, I felt suffocated, like I couldn’t breathe. I can’t save either my brother or my parents. They actually have to save themselves. It took a long time for this to fully sink in. I had a poor self-image for many years.

The last part of the story described the good years with more clarified and distanced relationships with family and significant support from her spouse. Gretha linked these years to her new understanding where she lost hope that the drug challenges would improve but found a way to be a sister who gave her brother great family experiences. She explained that she could understand her parents, but she did not need to take on the role of the helper anymore for her parent’s sake. Gretha had a strong sibling bond, where it was unthinkable for her not to have contact with her brother. She explained how important it was that her spouse could accept her complex family relationships and welcome her brother to their home.

Gretha’s story shows how different constructions of position and role impact experiences, actions, and identity. When her understanding of substance use problems and her role in the family changed, she was able to act differently. Gretha ended her story by emphasizing the need for family members living with ongoing drug use. She wished someone had contacted her when she was young and helped her not to take all that responsibility as a sibling.

### Theme 3: it’s worse for the parents

“It’s worse for the parents” is a theme about how the relationship with parents influences relationships with siblings with drug challenges. Participants relate how the choice to have contact with a sibling can be taken to relieve or protect parents, and not just for the sake of themselves. Sibling relationships with parents are highlighted in these stories, and siblings’ choices are linked to their stories about the whole family. Through retelling some of Tom’s story, we illustrate how the parent relationship can affect the relationship with a sibling with a drug use problem.

#### Tom’s story: the history of mother’s burden

The main characters in Tom’s story are his brother, who is three years older, and his mother. Tom links his own experiences of his brother’s drug use problems to the overwhelming impact on his mother.

She was there the entire time, right until the end, helping him. Mum helped him considerably financially. My guess is that she spent a million on him over the last 15 years. I suspect she has spent quite a lot of her money on my brother. There are plenty of things that need fixing at her house, but that has not been fixed because the money has not been there.

Tom tells a story of ambivalence and understanding. Tom presented his understanding of his mother’s choice to help his brother again and again, but he also said he believed that the help was not actually helping. He narrated that he understands why his mother prioritized the brother but also how it still hurt him. Tom explained how exhausting his brother’s problems have been for his mother or how impossible a choice his mother has faced. Tom related how his brother’s death was, for Tom, a great sadness but also a relief. He presented the understanding that if his brother had lived, he would have continued to use drugs. These thoughts linked Tom to his mother’s situation, and he explained that he was convinced that this had worn his mother out and caused misery for the whole family. Tom explained:

She should not have been put in the position to offer help. It’s possible that, in the long run, your love for your child makes it virtually impossible to make tough decisions. This had been going on for 15 years – the same situation over and over and over again. So, where would it end? It was very sad when he died but also a relief. I am fully convinced that, unless a miracle happened, he would’ve continued using for another 20 years. And that would’ve worn my mother out completely. For me, his death was a relief, without a doubt.

Tom linked his story to an understanding of different family roles, presented with his words like “there’s a difference between mom and me because he was her baby while I am a brother”. In his story, the brother’s challenges are presented as less demanding than the mother’s. Tom shared that he felt sad because of his brother’s situation, but he did not define himself as the one who could or wanted to influence his brother’s life choices. At the same time, Tom linked his actions to his understanding that this felt different for his mother. In Tom’s story, the mother’s feelings and choices are more important than Tom’s, even when his own needs are downgraded.

### Theme 4: ‘We’ as a synonym for the family

The theme of “we” as a synonym for the family reflects how relations in a family can help family members cope with the demanding life situation themselves and help the substance-using family member. It reflects how the challenges also can increase unity in the family. This theme presents how participants’ families went together through periods of ongoing drug use and drug-related death. Parts of William’s story are used to illustrate how families solved challenges, shared burdens, and supported each other. The brother’s drug use problems bound the family closer together in this story.

#### William’s story: the history of we

William related how his younger brother had drug use problems for many years, but it took time before the family understood the seriousness of these problems. William explained:

So, being a close relative of his for a very long time, we did not think of it as having a person with a serious drug problem in the family. We knew he was using, we knew he was drinking heavily, and we knew that he abused prescription drugs, but he was not really your typical drug addict.

William linked his story from the we-perspective. It was for “us,” the family; his brother is reaching out and asking for help. It was “we,” the family, who do not understand the seriousness. It was the “we”, the family, who agreed that “It was the right decision to take him in, take care of him and support him, rather than throwing him out into the cold, as tempting as it was at times to shake him a bit and tell him to clean up his act!”

Eventually, William said his partner also became part of the “we”. William stated that despite the strong “we” story, there were differences between the roles in the family. William explained how his family members developed a differentiation of roles and tasks, which they were satisfied with. William said that he was his brother’s trusted confidant, “his psychologist”. In William’s story, some things were presented as being easier to say to the brother first before it was communicated to the parents (as information about his sexual orientation). The assessment of what he did concerning his brother was also presented as being connected to what he experienced was best for the family. For example, William explained:

I don’t want to get stuck in a rut of wondering about what I could have done differently to save him, also when it comes to my mum and dad. I don’t want to say or do anything they might interpret as suggesting they should’ve done things differently.

William linked the end of his story to the “we” by telling how his brother’s death brought the family into a crisis, making family ties even closer. William described how the family had a new feeling of unity:

What happened was just horrible and threw the whole family into a crisis, but at the same time, when I look back on it now, I see how it drew our family closer. There is a sense of solidarity between the three of us who are still here, as well as my significant other, and we always give each other a hug when we meet, and we don’t take each other for granted because we know all too well that things can turn upside down in a matter of moments.

## Discussion

The four themes presented in this article demonstrated how complex and multifaceted siblings’ stories about living with their brothers or sisters’ ongoing drug use are. These four stories provide nuanced insight into how bereaved siblings piece together the meaning of the impact their siblings’ drug use problems had on their family relations. Results in this study are based on siblings’ retrospective versions, and some of them told their stories three years after losing their sibling. They are not discussing life with siblings’ problematic substance use situated in the middle of the problems but as bereaved siblings looking back to life before death. A consequence of interviewing siblings in retrospect about their experiences is that stories probably are modified and worked through over time and shaped by experiencing the death of the brother or sister. The loss of a sibling in drug-related death may have made it easier to talk about some experiences because the acute and ongoing strains and hopes are in the past. At the same time, it can also be harder to remember the difficult part because the good memories of siblings may have been given more space. For some participants, the period of living with siblings’ problems was long, and they looked back to events that took place several years ago. Some memories have surely disappeared, and others may be stronger years later. We have taken their narratives as personalized, situated and developed versions of life with siblings’ problematic substance use, produced and presented in the meaning-making occasion of the interview (Gubrium & Holstein, 2011) (emphasizing their present interpretations of the situation formed by bereavement). Acknowledging that experiences are transformed by time, we still assume that the stories told in the interview provide the potential for insight into how the participants give meaning to their sibling’s drug use problem.

This study supports previous findings of how siblings’ experiences of living with brothers or sisters’ drug use problems are closely linked to the whole family’s life situation. All the stories presented in this study reflect and link the sibling’s situation to the realities in the family. It is supported by Howard et al. (2010), who claims that being a sibling of a brother or sister with problematic drug use may change not only the relationship with that person but also the sibling’s relationship with parents, other siblings, and themselves. A sibling’s story may be a history of a shared upbringing in a multi-problem family or a lifelong struggle to distance from the assigned role in the family. It may also be the story of understanding and protecting parents, also against siblings’ own beliefs. It may also unfold as an experienced unity and connection in the family, which helps the individual family member and the family as a whole.

The serious consequences of problematic drug use for individuals seem to draw attention both from the person using drugs and the other family members, as well as professionals and other support providers (Lindeman et al., 2021). It can create a picture of families where problems arise because one of the children in the family develops drug use problems. The problems of the individual family member can also be one of the many challenges in the family or even a response to these challenges. As one of the participants in this study said, the struggle is about the whole living situation in some families. The need for help for the entire family has been present long before a child’s problematic drug use problems began. Previous research has clarified how familial, social, and individual risk factors increase the possibility of an individual developing a drug use problem (Whitesell et al., 2013). Vulnerability to drug use problems seems to be significantly heightened among individuals with a family history of drug use disorder (Cservenka, 2016). Social problems such as poverty, socioeconomic deprivation, unemployment, and familial problems are often present simultaneously (Saxena et al., 2006). There is no reason to idealize family life. Still, the family is often of great importance as a basis for people’s basic needs for love, security, belonging, care, and social development (Lorås & Ness, 2019). The current study’s findings show how the burdens could continue for generations. The sibling who had experienced familial problems in childhood continued to help their drug-using siblings’ children and grandchildren in bad childcare situations. These siblings’ situation requires attention and sensitivity from professionals. Our findings pointed out that siblings’ experiences of lacking help in childhood could result in scepticism towards and distrust of professional services.

Our findings show how siblings often understood their parents’ situation and feelings. This understanding sets norms for their actions. For example, siblings accepted this decision because siblings understood why it was difficult for their parents not to help their drug-using child. Sometimes siblings agreed with this decision, while at other times, they had contradictions themselves but accepted the parents’ choice. Siblings in this study acknowledged being the parent as a unique position in the family. Parents are both positioned as the most responsible and with the most tremendous burden. The parents’ love and care for the child were seen as unique and different from the sibling relationship. Also, research and social and healthcare practices often focus on the parents, and the position of siblings is often forgotten (Schmid et al., 2009; Smith-Genthôs et al., 2017).

The narrative analysis gives the possibility to increase the audibility of some stories (Frank, 2012). The current study has provided an opportunity to give this place to siblings’ stories, both as individual life stories and as the stories of siblings’ roles in family dynamics. The findings show how significant and far-reaching experiences for siblings could be. It shaped siblings as a person, and many of our participants spent years trying to find out who they wanted to be as individuals and as a part of the family dynamics. For some of them, these experiences provided learning and development of personal qualities that they appreciated, such as the experience of better self-esteem and more clarified family relationships. For others, it created relational challenges and insecurity, which affected their new relations, such as living with a partner. Given how defining these experiences could be for siblings, siblings’ stories must be heard and acknowledged by researchers and professionals. Unfortunately, it seems that siblings’ situations easily remain in the shadow of the struggles of their parents and their drug-using brother or sister.

Several earlier studies (Adams, 2008; Copello et al., 2010; Selbekk & Sagvaag, 2016) have problematized the individual-oriented perspectives in drug use services that provide limited opportunities for integrated work with families. The present study also shows what possibilities professionals lose when the family perspective is not included (such as acknowledging siblings’ needs and facilitating family conversations about difficult themes). Siblings in this study are involved and can have an in-depth understanding of their siblings’ and parents’ situation and their opportunities for action and choices. Siblings treated complex topics such as guilt and shame with care, not to burden the parents. We believe that facilitating family conversations where these potentially difficult topics could be addressed could be an essential part of breaking the silence and building down the problematic situation many families experiences. A review of studies examining substance use and family relations illustrates that families experience isolation and loneliness (Lindeman et al., 2021). Thematisation of topics such as guilt and shame in family conversations facilitated by professionals could prevent the isolation and loneliness families may experience.

## Strengths and limitations

As mentioned earlier, the current study has a retrospective perspective. All participants look back to the life phase before their sibling’s drug-related death. The retrospective perspective can be both a strength and a limitation of this study. Siblings may be more able to articulate the life situation they experienced, and their reflections may have clarified the story’s aspects. At the same time, some elements and tensions of the story may have been lost. We think that bereavement may change the story, but it is no less genuine and essential. Nevertheless, we acknowledge that more studies are needed to focus on the family situation both while siblings are alive and as siblings who have been bereaved after drug-related death.

The study with fourteen participants has strengths in the comprehensive and detailed interviews and the possibility of close reading of stories. Narrative analysis asks specific questions about particular lives in a specific context (Emerson & Frosh, 2004). This study’s strength is that it focuses on the nuanced and complex and, by doing that provides in-depth insight into the research topic of this study. At the same time, our study is limited in terms of filling the existing knowledge gaps about siblings’ experiences of living with a brother or sister with drug use problems. More research is needed on different families and cultures. All of the selected stories represent siblings who lived in the same household and were close in age to their drug-using siblings. It is important to keep in mind that not all siblings grow up in the same home or have the same genetic origin. We also call for more research on how structural variables, such as sibling age, gender, birth order, number of siblings, and family characteristics, impact siblings’ experiences. It is also important to understand if and how siblings’ experiences differ if their sister or brother has drug use problems compared to other types of challenges, such as psychological and somatic challenges and various disabilities. This knowledge is important for professionals to offer these siblings and their families the help they need.

## Implications for policy and practice

With presented stories and discussion, this study is practice-oriented and opens many implications for practice. The findings from our study hopefully contribute to an increased awareness of siblings’ life situations and needs. Adult siblings often prioritize parents’ support needs ahead of their own and despite having long-term consequences in their own lives, affecting their self-image and relationships. Younger siblings may be forgotten because the practitioners may have respect for the parent’s role as supporters for their children and may have too high expectations that parents will manage this task when they themselves are experiencing demanding life situations. The narrative focus in this article also shows what knowledge can come from listening to siblings’ meaning-making and how their life situations can provide a basis for quite different experiences and interpretations. We hope one implication and inspiration for practice is to notice the value of listening to siblings’ meaning-making. In conversations with siblings, practitioners can be in a position to contribute to developing stories that are helpful for siblings to give evidence to their experiences.

This study, as previous studies, shows clearly how siblings’ experiences of living with brothers or sisters’ drug use problems are closely linked to the whole family’s life situation, calling for family-oriented support. Siblings’ life situations are different, so family-oriented help can mean both comprehensive help for families where there are multiple problems but also periodic help in crises to prevent siblings’ experience of being forgotten in the family where brothers’ or sisters’ problems take parents’ attention. With a focus on medical perspectives, with service structures built for treatment for individuals, and where family involvement occurs to a limited extent (Kalsås et al., 2020; Selbekk & Sagvaag, 2016), relational perspectives have had little space in substance use services. This is not a new discussion, and Kalsås et al. (2020), for example, raise a strong recommendation that family and network-oriented work should be included in services as part of the treatment directed for individuals with substance use problems and for families. Our findings clearly show that siblings may be an especially hidden and overlooked population in a field where relational perspectives are lacking. Implications for practice from this study should be directed at politicians because the current organization of services in many countries creates little room for relational ways of working and lacks anchoring family perspectives in the framework of the services.

## Concluding comments

The findings of this study identified four different stories that shed light on various aspects of how bereaved siblings piece together meaning regarding the impact siblings’ drug use problems had on their family relations. In the four stories, siblings linked their experiences to their family situation. Stories highlighted how some siblings could experience the impact as a part of many problems in the family, while other siblings experienced even closer connections and unity in their families. Siblings’ drug use could have lifelong effects on participants’ lives and family relations.

This study calls for more attention to siblings’ situations. We would argue that researchers and professionals in the fields of drug use, social work, childcare, and family therapy need more awareness of how siblings’ experiences are complex and deeply rooted in their family situation. Siblings’ lives are affected by their siblings’ problems, and siblings should also be involved in routine support and treatment practices.

## Appendix I: Themes

**Table:**

	(1) Surviving difficult family life	(2) The relationships in continuous change	(3) It’s worse for the parents	4) “We”, as a synonym for the family
Lena	X			
Martin		X	X	
Susan		X		
William				X
Marthe	X			
Grethe		X		
Tom			X	
John	X	X		
Jane				X
Anna				X
Sarah	X			
Tina		X	X	
Evelyn			X	X
Hannah		X		X
